# Pathways of Neutrophil Granulocyte Activation in Hereditary Angioedema with C1 Inhibitor Deficiency

**DOI:** 10.1007/s12016-021-08847-4

**Published:** 2021-02-19

**Authors:** Erika Kajdácsi, Nóra Veszeli, Blanka Mező, Zsófia Jandrasics, Kinga Viktória Kőhalmi, Anne Lise Ferrara, László Cervenak, Lilian Varga, Henriette Farkas

**Affiliations:** 1 Research Laboratory, Department of Internal Medicine and Hematology, Semmelweis University, 46 Szentkirályi str, 1088 Budapest, Hungary; 2 MTA-SE Research Group of Immunology and Hematology, Hungarian Academy of Sciences and Semmelweis University, Budapest, Hungary; 3 Hungarian Angioedema Center of Reference and Excellence, Department of Internal Medicine and Haematology, Semmelweis University, Budapest, Hungary; 4 Department of Rheumatology, Hospital of Hospitaller Brothers of St. John of God, Budapest, Hungary; 5 Center for Basic and Clinical Immunology Research (CISI), Department of Translational Medical Science, University of Naples “Federico II”, Napoli, Italy

**Keywords:** Hereditary angioedema, Erythema marginatum, Neutrophils, Cytokines, C1-inhibitor, Bradykinin

## Abstract

Hereditary angioedema (HAE) with C1-inhibitor deficiency belongs to bradykinin-mediated angioedemas. It is characterized by recurrent subcutaneous and/or submucosal swelling episodes (HAE attacks) and erythema marginatum skin rash as a pre-attack (prodromal) phase. HAE attacks were shown to be accompanied by peripheral blood neutrophilia. We aimed to find molecular mechanisms that may explain the distinct role of neutrophil granulocytes in HAE. Plasma levels of blood cells and factors related to neutrophil activation (cytokines, chemokines, chemotactic factors, enzymes, and neutrophil extracellular trap) were measured in plasma samples obtained from patients during symptom-free periods (*n* = 77), during prodromal phase (*n* = 8) and attacks (*n* = 14), during a spontaneously resolved attack (*n* = 1), and in healthy controls (*n* = 79). Higher counts of white blood cells, lymphocytes, and neutrophil granulocytes were found in symptom-free patients compared with controls; these cell counts were elevated further during HAE attacks. The level of chemokine (C–C motif) ligand 5, monocyte chemoattractant protein-1, and myeloperoxidase were also higher in the symptom-free patients than in the controls. Levels of monocyte chemoattractant protein-1, leukotriene B4, neutrophil elastase, and myeloperoxidase were elevated during attacks. During erythema marginatum, white blood cells and monocyte count and levels of interleukin 8 were elevated compared with symptom-free period. Similar changes were detected during the attack follow-up. We conclude that the activation of NGs in symptom-free periods and a further increase observed during attacks suggests that NGs may be involved in the pathomechanism of HAE with C1-INH deficiency.

## Introduction

Hereditary angioedema (HAE) due to C1-inhibitor (C1-INH) deficiency (C1-INH-HAE) is a rare, potentially life-threatening disorder characterized by recurrent swelling episodes in subcutaneous and/or submucosal tissues, which are often preceded by prodromal symptoms [1, 2]. Forty-two to 58% of C1-INH-HAE patients have erythema marginatum (EM) as the only objective prodromal symptom of the HAE attacks [3, 4]. Mutations in the *SERPING1* gene results in decreased C1-INH function (type 1 low C1-INH, type 2 normal or increased C1-INH protein level) [5]. C1-INH is involved in the regulation of the complement, kallikrein-kinin, coagulation, and fibrinolytic plasma enzyme systems. The impairment of C1-INH function together with certain trigger factors can activate these cascade systems. The activation of the kallikrein-kinin system (KKS) results in cleavage of bradykinin (BK) from high molecular weight kininogen by plasma kallikrein (PKa). The release of BK increases vascular permeability, which causes angioedema by interacting with BK receptor B2 on the endothelium [6]. A number of articles have been published to explore the pathophysiology of C1-INH-HAE, focusing on the role of plasma enzyme systems and endothelial cells [7–9]. Increased white blood cell (WBC) count was observed during HAE attacks without apparent inflammatory signs. It was hypothesized that this is a consequence of hemoconcentration due to increased plasma extravasation of fluid into the extracellular space [10–12]. Zotter et al*.* observed that all hematologic values are increased during attacks and the elevation of WBC count is significantly greater (~ 1.5-fold) than expected based on hemoconcentration alone, and it involves specifically the peripheral blood neutrophil granulocytes (NGs) [13]. A further study showed that neutrophil granulocyte count (NGC) is higher in C1-INH-HAE patients during symptom-free periods than in healthy controls, and this difference is further emphasized during HAE attacks. Moreover, NGs were shown to be activated during edematous episodes [14]. It was proposed that release of NGs into the circulation contributes to the progression of HAE attacks, which may be precipitated by these cells themselves. NGs may contribute to the dysregulation of KKS, since this system can also be activated on the surface of neutrophils [15]. NGs are capable of producing cytokines and certain enzymes, and cytokines and chemokines produced by other cell types affect their functions. NGs produce neutrophil elastase (NE), myeloperoxidase (MPO), and proteinase 3 (PRTN3) in their azurophilic granules [16]. During activation of NGs, NE released from these cells may modify the C1-INH molecule and thereby further attenuate its already impaired function [17, 18]. MPO is a local mediator of tissue damage and the resulting inflammation in various inflammatory diseases [19, 20]. The primary biological function of PRTN3 depends on its proteolytic activity, which is the degradation of extracellular proteins at inflammation sites [21]. The activation of NGs may lead to the formation of neutrophil extracellular traps (NETs) during which NGs release their DNA content and citrullinated histones decorated with the proteins from azurophilic granules (e.g., NE and MPO), specific granules, tertiary granules, and the cytoplasm to bind pathogens and also provide a negatively charged surface for the activation of the KKS and the complement systems [22–26].

Moreover, activated endothelial cells and platelets may assist to the formation of NETs and the activation of NGs by producing pro-inflammatory cytokines. TNF alpha (TNFα), IL-8, and leukotriene B4 (LTB4) produced by NGs have autocrine effect on NG function itself [27, 28]. TNFα is a cytokine involved in acute phase reactions and systemic inflammation. It is a potent chemoattractant for NGs, inducing their migration to the tissues by promoting the expression of adhesion molecules on endothelial cells [29]. Furthermore, TNFα can induce IL-8 production from a variety of cells (e.g., endothelial cells, macrophages, neutrophils, and epithelial cells) [28]. IL-8 is a neutrophil chemotactic factor which induces chemotaxis, primarily of NGs to the site of infection [28, 30]. LTB4 also acts on NGs to elicit their chemotaxis and integrin-mediated adhesion to the vascular endothelium, thereby allowing them to bind to and cross into the tissue, and induces the formation of reactive oxygen species and the release of lysosomal enzymes [31].

There is limited data on levels of peripheral blood NGs and their function in C1-INH-HAE patients. Moreover, there is very limited data on the activation of these cells during HAE attack, or their activation during EM. The latter has not been investigated until now.

The objectives of our study were (1) to confirm our previous results, on higher NGC in patients compared with healthy controls, and elevated NGC in patients during attacks, as compared with symptom-free periods; (2) to find a molecular pattern among neutrophil chemoattractants and activation markers, which may explain the distinct behavior of NGs in HAE patients; and (3) to map the neutrophil function during EM and the kinetic changes during an HAE attack, followed from the beginning until its spontaneous termination.

## Study Population

### Patients

The patients were selected from the total C1-INH-HAE patient population (*n* = 197) receiving regular care at the Hungarian Angioedema Center of Reference and Excellence. The diagnosis of C1-INH-HAE was established by family history, evaluation of the clinical manifestations, and laboratory parameters (antigenic and functional levels of C1-INH, C1q, and C4). We included 77 consecutive patients who presented at the center for follow-up visits without HAE symptoms during 2014 and 2018, 14 patients with HAE attack, and 8 patients having prodromal (EM) without attack. A subcutaneous HAE attack was followed from the beginning until its spontaneous termination in one 56-year-old female patient with C1-INH-HAE type I [32]. None of the patients had any clinical manifestation suggestive of an acute infection at the time of blood sampling.

### Healthy Controls

**Fig. 1:**
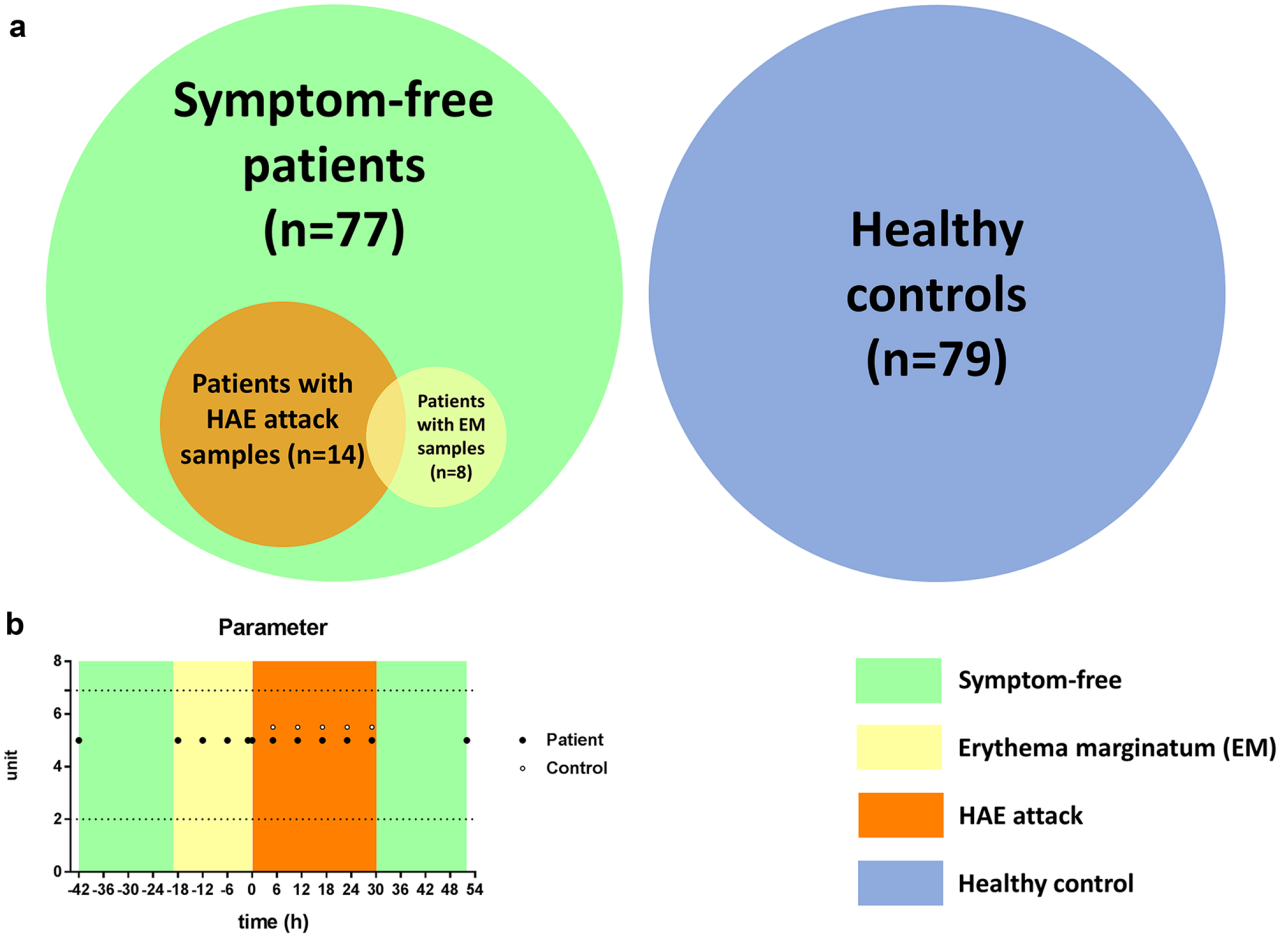
Disposition of subjects in the study. **a** We made three types of comparisons: controls (*n* = 79) with symptom-free patients (*n* = 77), symptom-free samples with the patients’ own attack samples (*n* = 14), and symptom-free samples with the patients’ own EM (prodromal) samples (*n* = 8). **b** A 56-year-old female patient with type I C1-INH-HAE was followed from her symptom-free phase (green), during EM (prodrome) (yellow) and angioedema attack (orange) until remission. Twelve EDTA-plasma samples were collected from the patient (solid circles) and 5 from the control person (open circles). Circles indicate sampling times. Normal range is represented by dotted lines

Healthy control group (gender and aged-matched) consisted of 79 healthy adults and one distinct gender- and aged-matched control for the HAE patient, who were studied in the kinetic follow-up part of the study. The healthy controls did not have any known medical diagnoses and did not receive medications at the time of blood sampling. C1-INH deficiency was excluded by complement testing (see above). Detailed demographic data of the patients and controls are summarized in Table 1 and Fig. 1 depicts the patient and control cohorts.

**Table 1:** Summary of the basic data of C1-INH-HAE patients and healthy controls

	Healthy controls (*n* = 79)	C1-INH-HAE patients		
		Symptom-free (*n* = 77)	HAE attack (*n* = 14)	EM prodrome (*n* = 8)
Male/female (*n*)	34/45	37/40	6/8	1/7
Age (years): median; 25–75% percentile	38; 32–46	41; 28.5–52	37.5; 27.3–49.3	40.5; 25.8–48.5
C1-INH-HAE type I/II (*n*)	-	70/7	11/3	8/0

## Methods

### Blood Sampling

Peripheral blood samples were obtained by venipuncture from patients with C1-INH-HAE during symptom-free periods, HAE attacks (before acute treatment), and EM, as well as from healthy subjects. Samples from the symptom-free periods were collected from patients who had been symptom-free for at least 72 h before blood sampling and had not received acute treatment for their HAE attack. The mean latency between the detection of the HAE symptoms and blood sampling was 7.8 h (SD: 7.6). The mean interval between symptom-free and during-an-attack sampling in the same patients was 9.5 months (SD: 7.7).

During the HAE attack kinetic follow-up part of the study, EDTA-plasma samples were collected 12 consecutive times, over 96 h of observation during an HAE attack in a single case. The patient rated the severity of her symptoms on a visual analog scale (VAS) More clinical details of this HAE attack have been described previously by Veszeli et al. [32].

EDTA plasma and serum samples were stored at − 80 °C until processing.

### Measurement of Blood Cell Count and Factors Related to Neutrophil Activation

WBC, NGs, lymphocyte, thrombocyte, monocyte, eosinophil, and basophil granulocyte counts were determined in the samples by using Advia120 Hematology System automate (Siemens, Erlangen, Germany). To determine the levels of the enzymes NE, MPO, PRTN3, as well as cytokines [TNF-α, IL-8, MCP-1, CCL5], and LTB4 levels, we used commercially available ELISA KITs. As negative control, we measured monocyte chemoattractant protein-1 (MCP-1) and chemokine (C–C motif) ligand 5 (CCL5) levels. MCP-1 is a chemoattractant and activating factor for monocytes, basophils, T cells, NK cells, and immature dendritic cells. MCP-1, in general, is considered as a chemotactic factor for monocytes and basophils but not neutrophils or lymphocytes are affected [34]. CCL-5 is a chemotactic stimulus for T cells, eosinophils, and basophils and plays an important role in attracting other types of leukocytes to inflammatory sites. The following ELISA KITs were used: IL-8 (KHC0083), NE (BMS269), and TNFα (KHC3014) from Thermo Fisher Scientific; LTB4 (KGE006B), CCL5 (DY278-05), and PRTN3 (DY6134-05) from R&D Systems; MPO (K 6631B) from Immundiagnostik; and MCP-1 (ab179886) from Abcam, according to the manufacturer’s instructions.

To measure NETs, we developed an in-house ELISA method. Briefly, anti-MPO antibody (PA5-16,672, Thermo Fisher Scientific) was used for capturing; for detection, we used anti-dsDNA antibody (ab27156, Abcam). The 96-well microtiter plates (Nunc® Maxisorp®) were coated with capture antibody 1:2000 overnight on 4 °C. After the blocking step (1 h room temperature, with 2% BSA-PBS), the standard and the samples (fourfold dilution) were incubated on the plate overnight on 4 °C. The next day, after washing the plate 3 times with PBS with 0.05% Tween 20, the detection antibody was incubated for 1 h on room temperature (1:4000). After washing 3 times, goat anti-mouse HRP-conjugated secondary antibody was used (1 h, room temperature, 1:2000, Southern Biotechnology). After 4 times washing, TMB substrate (Life Technologies) was used. The reaction was terminated with 1 M H_2_SO_4_, and OD was determined with a microplate reader (Infinite® M1000 PRO, Tecan Group Ltd) at 460/620 nm. The samples were calculated from the standard curve. The standard curve was generated from freshly isolated NGs from a healthy volunteer (10 × 10^6^ cells/2 ml HBSS + 20 mM HEPES buffer) activated with 100 nM PMA on 37 °C during a 3-h period. After incubation, the cells were centrifuged and the supernatant was collected and used as NET standard in 1/500 dilution, followed by 2 times linear dilution.

All laboratory tests were performed on freshly thawed plasma samples. All samples were measured in single assessments (with the re-measuring of some samples).

The study protocol was approved by the institutional review board of Semmelweis University of Budapest, and informed consent was obtained from all the participants in accordance with the Declaration of Helsinki.

### Statistical Analysis

The results of ELISA measurements were analyzed and interpreted using GraphPad Prism v7.00 (GraphPad Software Inc.). For statistical analysis, Mann–Whitney test, unpaired *t* test, paired *t* test, and Pearson’s *r* correlation test were used.

## Results

The main purpose of this study was to investigate the levels of peripheral blood NGs and the parameters related to their activation in C1-INH-HAE.

### Peripheral Blood Cell Counts and Plasma Levels of Factors Related to Neutrophil Activation in C1-INH-HAE Patients and Healthy Controls

Total WBC and different blood cell type counts were compared in peripheral blood samples obtained from 79 healthy subjects and 77 C1-INH-HAE patients. Total number of WBCs, lymphocytes, NGs, and basophil granulocytes was significantly higher in symptom-free C1-INH-HAE patients than in healthy controls (Table 2).

**Table 2:** Peripheral blood cell type count in samples obtained from healthy subjects and C1-INH-HAE patients during symptom-free periods

	Healthy controls (*n* = 79)	C1-INH-HAE symptom-free period (*n* = 77)	*P* value	Normal range
White blood cell (10^9^/L)	6.38 (5.1–7.4)	7.25 (6.3–8.5)	0.0013	4.00–10.00
Lymphocyte (10^9^/L)	1.92 (1.5–2.3)	2.14 (1.9–2.6)	0.0010	1.00–4.00
Thrombocyte (10^9^/L)	244.5 (215.3–286.8)	262.5 (224–306)	ns^a^	150–400
Monocyte (10^9^/L)	0.35 (0.28–0.42)	0.35 (0.3–0.43)	ns	0.15–0.90
Neutrophil granulocyte (10^9^/L)	3.78 (2.82–4.82)	4.34 (3.57–5.5)	0.0029	2.00–6.90
Eosinophil granulocyte (10^9^/L)	0.125 (0.04–0.18)	0.13 (0.08–0.16)	ns	0.03–0.50
Basophil granulocyte (10^9^/L)	0.03 (0.01–0.04)	0.04 (0.02–0.05)	0.0123	0.01–0.20

*Ns* non-significant

Values are medians (25–75% percentile). *P* values were calculated with Mann–Whitney test in most cases

^a^Unpaired *t* test

The neutrophil-related factors: CCL5, MCP-1, and MPO were significantly higher in patients during symptom-free periods than in healthy controls (Table 3).

**Table 3:** Factors related to neutrophil activation in healthy controls and C1-INH-HAE patients during symptom-free periods

	Healthy controls (*n* = 79)	C1-INH-HAE symptom-free period (*n* = 77)	*P* value
PRTN3 (ng/ml)	19.7 (14.6–28.69)	23.2 (16.2–27.8)	ns
CCL5 (ng/ml)	12.4 (5.1–24.7)	19.2 (10.2–28.7)	0.0076
TNF a (pg/ml)	12.4 (8.1–14.8)	9.9 (8.1–14.1)	ns
IL-8 (pg/ml)	0.94 (0.41–1.66)	1.01 (0.6–1.43)	ns
MCP-1 (pg/ml)	54 (44.6–73.7)	64.5 (52–83.2)	0.0288
NE (ng/ml)	31.6 (24.5–40.9)	29.9 (22.6–37.6)	ns
NET (activated cells/100 µl)	159.3 (132.4–191.4)	158.7 (139.2–190.9)	ns
MPO (ng/ml)	72.6 (54.6–90.1)	77.4 (64.4–102.1)	0.0478
LTB4 (ng/ml)	61.5 (45.1–110.3)	63 (41–158.1)	ns

*Ns:* non-significant

Values are medians (25–75% percentile). *P* values were calculated with Mann–Whitney test

### Peripheral Blood Cell Counts and Factors Related to Neutrophil Activation in C1-INH-HAE Patients During EM and HAE Attacks

Out of 77 C1-INH-HAE patients, plasma samples were collected from 8 patients during EM and from 14 patients during HAE attacks (Fig. 1). The body locations of the HAE attacks were subcutaneous in 8 patients, abdominal in 3 patients, and combined subcutaneous and abdominal in 3 patients. Peripheral blood cell types and neutrophil-related parameters in these 2 subgroups were compared:

WBC and monocyte cell counts were significantly elevated during EM (WBC: 8.47 10^9^/L ± 1.29, monocytes: 0.5 10^9^/L ± 0.16) compared with symptom-free periods (WBC: 7.21 10^9^/L ± 1.22, monocytes: 0.4 10^9^/L ± 0.13) (*p* = 0.0272 and *p* = 0.0169, respectively, paired *t* test). Among the neutrophil-related parameters, only the level of IL-8 was elevated during EM (1.66 pg/ml ± 0.93) compared with symptom-free period (0.88 pg/ml ± 0.30), (*p* = 0.0481, paired *t* test). The results are presented as means and ± SD.

**Fig. 2:**
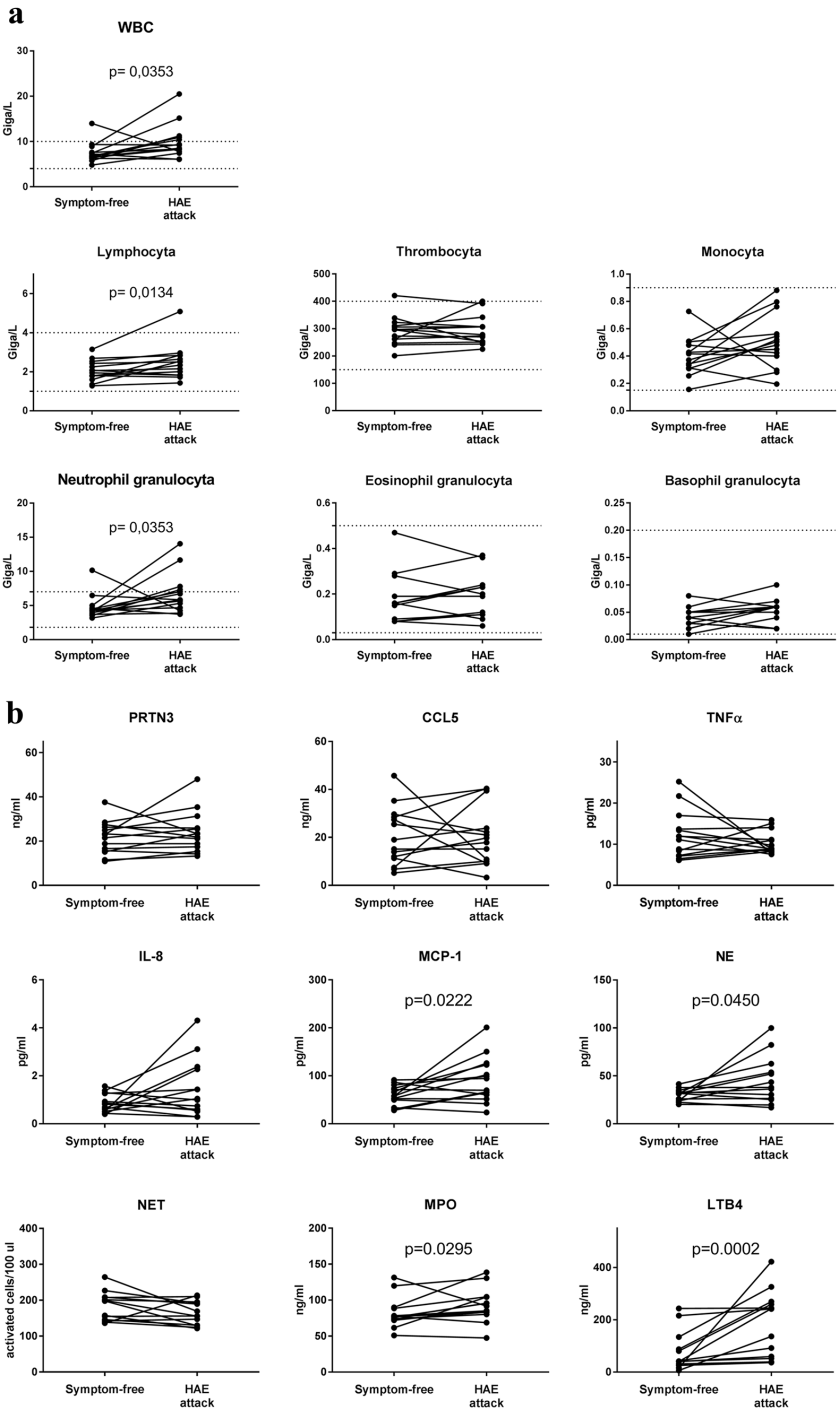
Blood cell count and factors related to neutrophil activation in HAE attacks compared with symptom-free periods. **a** *WBC, thrombocyte, lymphocyte, and neutrophil-, eosinophil-, and basophil granulocyte* count were measured in peripheral blood samples obtained from 14 patients in symptom-free periods and during HAE attacks. The dotted lines indicate normal range. Wilcoxon test (WBC, lymphocyte, neutrophil-, and eosinophil granulocyte) and paired *t* test (thrombocyte, monocyte, basophil granulocyte) calculations were used. **b** Levels of *neutrophil related factors* were measured from plasma samples of 14 patients in symptom-free periods and during HAE attacks. Wilcoxon test (TNFα, IL-8, MPO, LTB4) and paired *t* test (PRTN3, CCL5, MCP-1, NE, NET) were used

WBC, lymphocytes, and NGs count were significantly elevated during HAE attacks (Fig. 2a) compared with symptom-free periods. Additionally, neutrophil-related factors: MCP-1, NE, MPO, and LTB4 levels were significantly elevated during HAE attacks compared with symptom-free periods (Fig. 2b). Furthermore, we found correlations between the NGC and levels of PRTN3 (*r* = 0.7066, *p* = 0.0047), CCL5 (*r* = 0.5968, *p* = 0.0243), MCP-1 (*r* = 0.749, *p* = 0.0021), NE (*r* = 0.9353, *p* < 0.0001), and NET (*r* = 0.7626, *p* = 0.0022).

### Kinetics of Peripheral Blood Cell Counts and Factors Related to Neutrophil Activation During HAE Attack

**Fig. 3:**
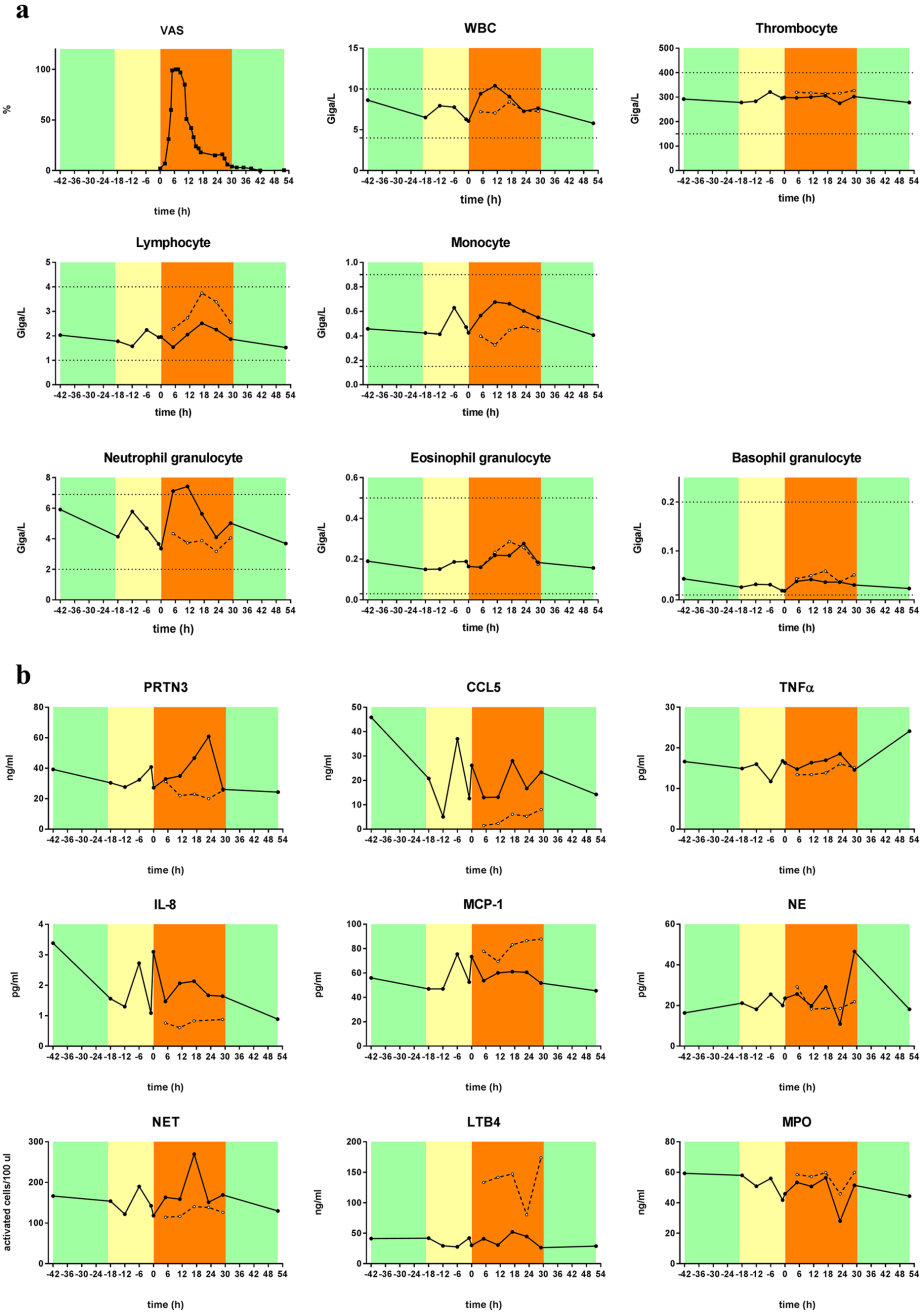
Kinetics of blood cell counts and factors related to neutrophil activation during an HAE attack (single patient). **a** Kinetics of *WBC, thrombocyte, lymphocyte, and neutrophil-, eosinophil-, and basophil-granulocyte* count during an HAE attack of a C1-INH-HAE patient (solid line with closed dots), and an age- and gender-matched healthy control (dashed line with open dots). Severity levels of angioedema symptoms were assessed by the patient in on a VAS scale (0–100 mm). Symptom-free periods are marked with green, prodromal phases with yellow, and attack phases with orange. The dotted lines indicate normal ranges. **b** Kinetics of *neutrophil-related factors* of the same patient and control

WBC and NG cell counts were elevated in one patient observed all through an HAE attack concurrent with severity of symptoms, as indicated by VAS score, 6–12 h after the beginning of the attack. During the follow-up, we observed two peaks of monocyte cell count, one during prodromal phase (characterized by EM) and the other during the attack phase (Fig. 3a). Neutrophil-related factors: IL-8, NET, and PRTN3 showed also a distinct peak during the prodromal (EM) phase and 18- and 24 h after the beginning of the attack, respectively (Fig. 3b).

## Discussion

Besides being the frontline cellular arm of the innate immunity against bacteria and fungi, NGs are able to actively regulate certain plasma enzyme cascade systems by producing and activating their components, as well as by forming NET as a negatively charged surface [33]. This is also true contrariwise: activation of plasma enzyme cascades significantly regulates the function of NGs at multiple levels. Therefore, we aimed to map the role of NGs in C1-INH-HAE, where the disturbance of plasma enzyme cascade systems (i.e., KKS, complement, fibrinolysis) is known. For better understanding, we summarized the between-group differences in Table 4.

**Table 4:**
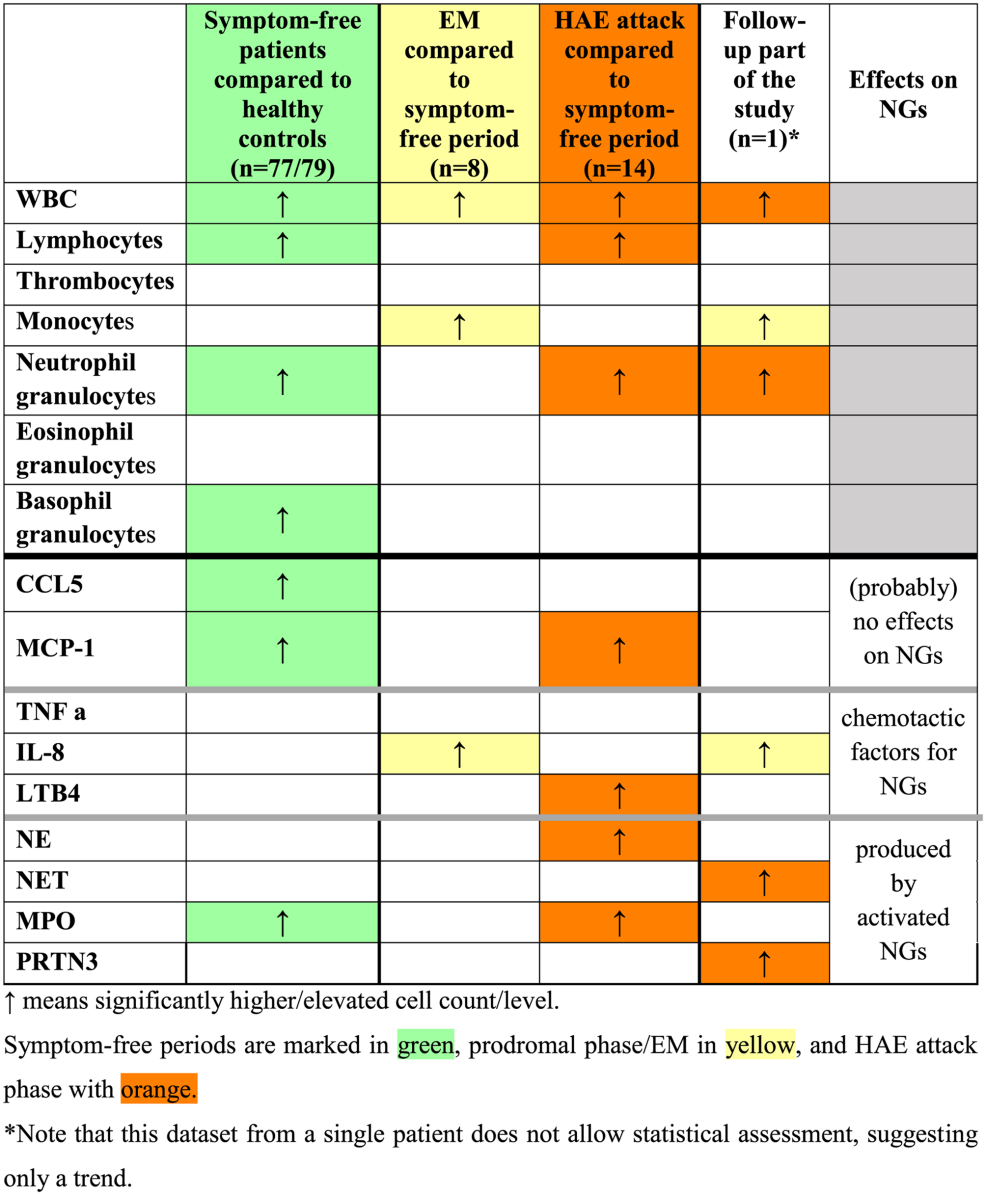
Between-group difference of the measured cell counts and levels of factors related to neutrophil activation

### Cellular Alterations

In C1-INH-HAE patients, an elevated WBC count was found during the prodromal (EM) and HAE attack phases; the composition of this cell population was different in the two phases. EM was characterized predominantly by elevated monocyte count, whereas HAE attacks NGC were elevated, as compared with symptom-free periods. Stress and physical exercise are known trigger factors of increased total WBC and NG counts as well as HAE attacks [34–36]. We cannot exclude that the increased WBC and NGC were caused by these trigger factors during the HAE attacks. However, elevated NGC and WBC were also found in symptom-free patients compared with healthy controls, which may suggest a stress or physical exercise independent elevation of cell counts at least in symptom-free period.

Elevated lymphocyte count was observed, in symptom-free patients compared with controls and also during HAE attack compared with symptom-free periods. Furthermore, monocyte count was also elevated during EM compared with symptom-free periods (also in the kinetic follow-up part). However, since our present study focused on the role of NGs in C1-INH-HAE, these results can be the basis for a future research.

### Chemotactic Factors

Only two of the chemotactic factors that have effects on neutrophil cells are found elevated: IL-8 and LTB4. IL-8 was elevated in C1-INH-HAE during prodromal (EM) phase compared with symptom-free periods, and we could see the same elevation in the case of IL-8 in the kinetic follow-up part’s prodromal phase that we have seen in the case of monocyte count. Monocytes are capable of producing IL-8, so we may assume that the elevation of these two factors be related. Monocytes are capable of producing IL-8 upon physical contact with endothelial cells, and this response is further augmented by pretreatment of endothelial cells with interferon gamma [37, 38]. Therefore, it is possible that elevated monocyte count and/or the interaction between monocytes and endothelial cells are accounted for the elevated IL-8 level during prodromal EM. IL-8 also plays a key role in NG recruitment and degranulation [39]. Therefore, this may suggest that these alterations are initiated at an early pre-attack (prodromal) stage and continue into the clinical attack.

Plasma LTB4 was also elevated during attacks compared with symptom-free periods. LTB4 enhances the adhesion of the leukocytes to the endothelium—reinforced by activated endothelial cells [40]—and may potentiate NGs to attach and migrate into the tissues. This process seems to contradict the increased NGC in the blood. We do not know how many NGs bind to endothelial cells or how many migrate and become sequestered in the tissues, but the elevated plasma levels of LTB4 raises the possibility that we underestimate the levels of NGs and its activation during HAE attacks.

### Activated NG Products

From the factors produced by activated NGs, all four were found elevated. MPO level was higher in symptom-free patients compared with healthy controls and was also elevated further during HAE attacks. Thus, in symptom-free periods, in spite of the higher NG numbers, their activation is only partially expressed. During HAE attacks, further elevation of the MPO level, parallel with elevation of NGC, occurred, and the level of NE was also elevated compared with symptom-free periods. In the kinetic follow-up part of the study, levels of PRTN3 and NET were elevated only during HAE attack period. All these results and the strong correlation found between NGC and PRTN3, CCL5, MCP-1, NE, and NET further support that NGs are activated during HAE attacks. Activation of the KKS can occur on the surface of NGs and NET [15, 25, 26]. On the other hand, NGs can be activated by kinins formed during inflammation [41]. In a previous study [42], elevated kallikrein/C1-INH complexes were found in C1-INH-HAE patients during symptom-free periods compared with healthy controls and also at the onset of HAE attacks (kinetic follow-up part). This suggests a correlation between activation of NGs and activation of the KKS. However, the question emerges, whether the NGs or its NET product induce KKS activation during HAE attacks, or conversely, the activation of the KKS attracts and activates NGs.

### Other Factors

Both CCL5 and MCP-1 levels were higher in symptom-free patients compared with healthy controls, and MCP-1 levels was further elevated during HAE attacks. These results suggest that CCL5 and MCP-1 may also act on NGs and could explain the higher/elevated NG levels in C1-INH-HAE patients. Moreover, Balamayooran et al. found that MCP-1 has direct and indirect effects on NG migration in mice [43], and a similar result was found in the relation of CCL5 by Stefano et al. in chronic obstructive airway disease (COPD) patients [44].

### The Contribution of the Study

There is relatively limited data about NGs and factors related to neutrophil activation in C1-INH-HAE patients. We propose that the higher level of NGs in the symptom-free stage of the patients may contribute to the constant activation of the KKS [42]. In contrast to previous studies [14, 45], we could not find any significant difference in IL-8 levels—one of the best-known chemotactic factors for NGs—between patients and healthy controls. Interestingly, we found that CCL5 and MCP-1 may have an important role in NG recruitment in HAE. Furthermore, we succeeded in adding a novel aspect: recruitment of the NGs during the early, prodromal (EM) phase [3, 46, 47]—concurrently with elevated monocyte count and elevated IL-8 level, which presumably originate from monocytes. Our observation supports the hypothesis that EM is an early phase of an HAE attack. This might provide an objective basis for new, individualized therapeutic strategies, such as administering acute treatment for HAE as early as the prodrome [3, 46].

During HAE attack, NGC increases, and NG activation also takes place, which can further enhance the KKS activation to maintain/contribute to the increased endothelial permeability. Moreover, NGs and endothelial cells may also have a (pre)activated state that promotes the adhesion [40],

### Weaknesses of the Study

The increased number of NGs and activation might have been underestimated. Our result refers only to the processes taking place in the circulation; thus, the confirmation of the pathogenic role of NGs in the tissues during HAE attacks requires further investigation. We had limited (8 and 14 samples) from patients during EM and HAE attack, respectively. Despite of the unique approach, we have just one patient with one attack in the attack kinetics follow-up part.

This study was focusing on NGs as supporting cells in the pathomechanism of C1-INH-HAE. The finding has brought to light new findings but raises questions as well. Apparently, C1-INH-HAE pathology is more complex than thought before. It raises a “chicken or egg” causality dilemma: are the NGs activated first and causing local edema, or the edema processes activates NGs?

## Conclusion

The alterations of NGC found in C1-INH-HAE patients may be driven by several factors, depending on the genotype, environmental conditions, nature of the triggering factor, and the actual molecular processes of the pathogenesis. These observations strongly support the hypothesis that C1-INH-HAE has a multifactorial pathomechanisms, beyond the monogenetic mutation in *SERPING1* gene*.*

## Abbreviations

BK: Bradykinin
C1-INH: C1 esterase inhibitor
C1-INH-HAE: HAE with C1-INH deficiency
C1q, C3, C4: Complement components
CCL5: Chemokine (C–C motif) ligand 5
COPD: Chronic obstructive airway disease
HAE: Hereditary angioedema
EM: Erythema marginatum
KKS: Kinin-kallikrein system
LTB4: Leukotriene B4
MCP-1: Monocyte chemoattractant protein-1
MPO: Myeloperoxidase
NE: Neutrophil elastase
NET: Neutrophil extracellular trap
NGC: Neutrophil granulocyte count
NGs: Neutrophil granulocytes
PKa: Plasma kallikrein
PRTN3: Proteinase 3
TNFα: Tissue necrosis factor alpha
VAS: Visual analog scale
WBC: White blood cell
